# Can Inpatient Hospital Experiences Predict Central Line-Associated Bloodstream Infections?

**DOI:** 10.1371/journal.pone.0061097

**Published:** 2013-04-05

**Authors:** Daniel M. Saman, Kevin T. Kavanagh, Brian Johnson, M. Nawal Lutfiyya

**Affiliations:** 1 Essentia Institute of Rural Health, Duluth, Minnesota, United States of America; 2 Health Watch USA, Somerset, Kentucky, United States of America; University of Maryland, School of Medicine, United States of America

## Abstract

**Background:**

Factors that increase the risk of central line-associated bloodstream infections (CLABSIs) are not fully understood. Recently, Hospital Compare began compiling data from hospital-required reporting to the CDC's National Healthcare Safety Network on CLABSIs in intensive care units (ICUs), at over 4,000 Medicare-certified hospitals in the United States, and made this data accessible on a central website. Also available on the same website are results from the Hospital Consumer Assessment of Healthcare Providers and Systems survey of patients' hospital experiences. Utilizing both databases, our objective was to determine whether patients' hospital experiences were significantly associated with increased risk for reported ICU CLABSI.

**Methods and Findings:**

We conducted a zero-inflated Poisson regression analysis at the hospital level on CLABSI-observed cases by ICUs in acute care hospitals (n = 1987) in the United States between January 1, 2011, and December 31, 2011. During this period there were a total of 10,866 CLABSI cases and 9,543,765 central line days. In our final model, the percent of patients who reported that they “sometimes” or “never” received help as soon as they wanted was significantly associated with an increased risk for CLABSIs.

**Conclusions:**

Using national datasets, we found that inpatients' hospital experiences were significantly associated with an increased risk of ICU reported CLABSIs. This study suggests that hospitals with lower staff responsiveness, perhaps because of an understaffing of nurse and supportive personnel, are at an increased risk for CLABSIs. This study bolsters the evidence that patient surveys may be a useful surrogate to predicting the incidence of hospital acquired conditions, including CLABSIs. Moreover, our study found that poor staff responsiveness may be indicative of greater hospital problems and generally poorly performing hospitals; and that this finding may be a symptom of hospitals with a multitude of problems, including patient safety problems, and not a direct cause.

## Introduction

Healthcare associated infections (HAIs) are one of the top ten causes of death in the United States [1]. A national reporting system, the National Healthcare Safety Network (NHSN) is currently operated by the Centers for Disease Control and Prevention (CDC) for the reporting of infections. Although most reporting is voluntary, the continued epidemic of HAIs has spurred a mandatory reporting system for several types of infections including methicillin-resistant *Staphylococcus aureus* (MRSA) bloodstream infections, *Clostriduim difficile* infections, surgical site infections (SSIs), catheter associated urinary tract infections (CAUTIs), and central line-associated blood stream infections (CLABSIs) [2]. Currently, data from the nationwide mandatory reporting initiative is only available for CLABSIs in intensive care units (ICUs). Other infections are planned to be reported online in the ensuing years.

In 2007, a report by the CDC on the medical costs of HAIs found that there were 92,011 CLABSIs in U.S. hospitals at an annual cost of between $0.67 to $2.68 billion dollars [3] and a case fatality rate of up to 25% [4]. Currently, the CDC estimates that there are 41,000 CLABSIs which occur each year in U.S. hospitals [5]. However, CLABSIs have been documented to be decreasing in the ICU setting, from 43,000 cases in 2001 to 18,000 cases in 2009 [4]. Implementation of prevention protocols and checklists, as described by Berenholtz, et al. (2004) [6], can decrease the incidence of CLABSIs in facilities up to 66% [7], [8]. However, these protocols have not been uniformly adopted in all facilities; a large number of facilities have achieved a CLABSI rate close to zero while in others it remains very high [9].

The prevention of CLABSIs requires following a strict sterile technique when a catheter is inserted and proper care and maintenance of the catheter site while the line is in place. This outcome is dependent upon an entire team working together and the continued availability of nursing care to maintain the catheter [10]. Furthermore, proper maintenance of the catheter site may also depend on adequate hospital staff, hospital cleanliness, and perhaps even proper communication with patients (i.e., so patients do not disturb the catheter site).

A possible reason for the slow adoption of patient safety protocols is often referred to as difficulty in changing a facility's staff or administration's culture. For the facility's staff this implies a resistance to change even in the presence of adequate knowledge and resources for this change [11]. However, for the administration, this implies a cost-driven system where improvement measures in patient safety may be especially difficult to implement during times of financial stress. Thus, improving patient safety requires a healthy staff working environment, an adequate nurse-to-patient ratio at the facility, and even an awareness of nurse burnout [11], [12], [13].

Customer surveys have been used to improve and develop products in almost every industrial sector in the United States. In healthcare, the adoption of patient surveys to guide the industry and for use in value-based purchasing is in its infancy. The Hospital Consumer Assessment of Healthcare Providers and Systems (HCAHPS) survey is currently used by the Centers for Medicare and Medicaid Services (CMS) for their pay-for-performance initiatives. It has been observed that the nurse working environment was significantly related to all HCAHPS measures and nurse workloads were related to patient's ratings of the institution and satisfaction with discharge information [14]. The Institute of Medicine also observed that “Patients' reports of satisfaction are higher in hospitals where nurses practice in better work environments or with more favorable patient-to-nurse ratios“ [15].

Thus, the metrics used in the HCAHPS survey may serve as surrogates for overall patient safety culture in an institution and as such may be highly correlated to a number of other safety measures. Three metrics were chosen for study:

- The metric for room cleanliness was chosen since intuitively it is the one which would be expected to have a large impact on infection rates.
- The metric for responding immediately was chosen since it is commonly used for a surrogate for a proper nurse working environment and staffing levels, both by The Joint Commission and CMS. So important is this metric, that regulations for the Conditions of Participation with Medicare require that when needed, a registered nurse is required to be immediately available for the bedside care of any patient [16].
- A third metric, communication between the patient and nursing staff, is an important safety metric. This metric is used as a major measure by Consumer Reports in their hospital rankings [17], [18]. This metric has also been observed to have a significant impact on facility readmission rates [19].

Dixon-Woods, et al. (2011) observed that one of the factors to achieve a reduction in CLABSIs was the shaping within an institution of “a culture of commitment to doing better in practices“ [20]. It is the purpose of this study to evaluate post-discharge patient surveys using HCAHPS data to determine what factors lead to an increased risk of reported CLABSIs in hospital ICUs. The results will add further evidence regarding patient surveys as a predictor of the safety culture at a facility and have immediate implications to help hospitals reduce their rate of CLABSIs and create safer patient environments.

## Methods

This research was approved by the Essentia Health Scientific Review Board (SRB).

### Data sources

Three data sets were used in this analysis (Table 1). First, we used data from Hospital Compare which contains CLABSIs reported to the NHSN from January 1, 2011, to December 31, 2011 [21]. This dataset was derived from the Centers of Medicare and Medicaid (CMS) mandatory reporting initiative for CLABSIs in ICU locations. Facilities from all 50 states plus Puerto Rico and the District of Columbia reported data. Out of 3617 hospitals, 1987 facilities were included in the analysis, 1120 facilities had no data available because their predicted number of CLABSIs was less than one, and 510 facilities did not have an ICU location. To be included in the NHSN dataset, a facility's predicted (or expected) number of CLABSI—an estimate based on infections reported to NHSN during January 2006 to December 2008 [22]—had to be greater than or equal to one [23]. From this dataset we used three variables: CLABSI observed cases, standardized infection ratio (SIR), and central line days by hospital.

**Table 1:** Dependent and independent variable data source.

*Data source*	*Variables*	*Sub parts of variables*	*Data collection dates*
Hospital Compare - Healthcare Associated Infections	Aggregate state level SIRs for CLABSIs	-	1/1/2010–12/31/2010
	CLABSI Observed Cases (dependent variable for ZIP model)	-	1/1/2011–12/31/2011
	CLABSI Central Line Days (ln central line days = offset term) (ZIP model)	-	1/1/2011–12/31/2011
	Hospital level SIR (dependent variable for linear regression model)	-	1/1/2011–12/31/2011
Hospital Consumer Assessment of Healthcare Providers and Systems (HCAHPS)	Percent of patients who reported that their nurses “sometimes” or “never” communicated well. (independent variable)		1/1/2011–12/31/2011
	Communication with Nurses (Composite measure)	Q1 - “During this hospital stay, how often did nurses treat you with courtesy and respect?” (never, sometimes, usually, always)	
		Q2- “During this hospital stay, how often did nurses listen carefully to you?” (never, sometimes, usually, always)	
		Q3- “During this hospital stay, how often did nurses explain things in a way you could understand?” (never, sometimes, usually, always)	
	Percent of patients who reported that they “sometimes” or “never” received help as soon as they wanted. (independent variable)		1/1/2011–12/31/2011
	Responsiveness of Hospital Staff (Composite measure)	Q4- “During this hospital stay, after you pressed the call button, how often did you get help as soon as you wanted it?” (never, sometimes, usually, always, I never pressed the call button)	
		Q11- “How often did you get help in getting to the bathroom or in using a bedpan as soon as you wanted?” (never, sometimes, usually, always)	
	Percent of patients who reported that their room and bathroom were “sometimes” or “never” clean. (independent variable)		1/1/2011–12/31/2011
	Cleanliness of Hospital Environment (Individual measure)	Q8- “During this hospital stay, how often were your room and bathroom kept clean?” (never, sometimes usually, always)	

*Note:* All hospitals were matched based on Medicare Provider ID Number and hospital names as a quality assurance.

We also used data from the Hospital Consumer Assessment of Healthcare Providers and Systems (HCAHPS) dataset found on the Hospital Compare website [24]. Three variables were matched by hospital Medicare ID number to hospitals in the NHSN dataset: patient communication with nurses, hospital staff responsiveness, and cleanliness of the hospital environment (Table 1). The HCAHPS is a survey of patients' hospital experiences administered to a random sample of adult hospital patients between 48 hours and six weeks after hospital discharge. This data is adjusted for patient mix and survey mode (mail only, telephone, mixed) by CMS before publicly reported on the Hospital Compare website [25]. In our dataset, 97% of hospitals had 300 or more completed surveys of discharged inpatients, which translates to at least 580,500 survey respondents across all hospitals in the NHSN dataset (*n* = 1987). The average survey response rate among hospitals in the NHSN dataset was 30.6% (SD = 7.5, Q1–Q3: 27%–35%).

A third data set was obtained from the CDC NHSN which was comprised of CLABSIs reported in 2010 from 2382 facilities from 41 states and the District of Columbia [26]. This data set was collected before the federal mandatory reporting initiative and was used to evaluate the effect of state reporting initiatives on the rate of CLABSIs. The average SIR was compared between states with high and low rates of reporting to help determine if state mandatory reporting laws introduced a significant variable.

### Study design and regression analysis

This epidemiological study utilized cross-sectional survey results, and infectious disease data on CLABSIs that occur in ICUs, with the unit of analysis being United States urban acute care hospitals (*n* = 1987). The outcome of interest in this modeling was count data, reflecting observed CLABSI cases by hospital ICU. Bivariate associations of the independent variables with the outcome were assessed using zero-inflated Poisson regression modeling. Pearson's correlation coefficients were also used to determine whether independent variables were highly correlated. Independent variables that were significantly associated with the outcome (p<0.05) were considered in the multivariate modeling process. The modeling process started with a full model including all independent variables. Only one variable remained in the final model as the addition of the other variables did not result in any meaningful change in the most significantly associated parameter estimate. Variable selection was also assessed utilizing the Akaike information criterion (AIC), where a lower AIC was indicative of better model fit. We utilized a zero-inflated Poisson (ZIP) regression model to fit the data and to account for the amount of hospitals with zero CLABSIs (*n* = 465, 23.4%). ZIP regression analyses were performed in SAS v.9.3 (SAS Institute, Cary, NC, USA) [27] using *proc genmod*, with distribution of ZIP, the natural log of central line days as the offset term, and the zero model including only the variable central line days, as we hypothesized that that was best variable to predict whether a hospital had zero versus at least one observed CLABSI case. Three continuous variables (communication with nurses, responsiveness of hospital staff, and cleanliness of hospital environment) were used in the modeling (Table 2). The dependent variable—observed CLABSI cases by hospital—was standardized by the number of central line days at each hospital and used as an offset term (where its beta coefficient was locked to the value of one during estimation). Although the dependent variable is the number of observed CLABSI cases, the model output parameter estimates can be understood to be controlled for central line days, rendering the dependent variable interpretation as observed cases per central line days [28], [29]. The count models' exponentiated beta coefficients were interpreted as adjusted estimated rate ratios, the zero models' exponentiated beta coefficient was interpreted as an estimated odds ratio, and 95% confidence intervals (CIs) were estimated.

**Table 2:** Descriptive statistics of hospital CLABSIs, central line days, and dependent variables.

*n = 1987 hospitals*					
ZIP Regression	Mean (SD)	Median	Q1 - 25%	Q3 - 75%	Range
*Dependent variable*					
Observed CLABSI cases (including zeros)	5.47 (9.28)	2	1	6	0–105
Observed CLABSI cases (excluding zeros)	7.15 (10.02)	3	2	8	-
Zero CLABSI cases n(%), 465(23.4)	-	-	-	-	-
*Certain Zero Group independent variable (zeromodel)*					
CLABSI central line days	4803 (5876)	2781	1437	5545	373–61359
*Independent variables (continuous)*					
Percent of patients who reported that they “sometimes” or “never” received help as soon as they wanted	12.13 (4.92)	11	9	14	0%–42%
Percent of patients who reported that their room and bathroom were “sometimes” or “never” clean	10.25 (3.76)	10	8	12.5	0%–31%
Percent of patients who reported that their nurses “sometimes” or “never” communicated well	5.70 (2.83)	5	4	7	0%–42%
**Linear Regression Model**					
*Dependent variable*					
Standardized infection ratio (SIR)	0.57 (0.60)	0.44	0.11	0.82	0–5.1

As a sensitivity analysis, and because the SIR is adjusted for both patient and facility characteristics and the HCAHPS survey is adjusted for patient mix, we also performed a linear regression analysis using the SIR as the dependent variable to confirm the results of the ZIP analysis. Though the ZIP analysis is the primary analysis, both regression analyses were performed because the SIRs validity as a measurement has recently been called into questioned [9], [30]. For example, the SIR adjusts for facility characteristics that include size and teaching institution. Both of these variables are highly correlated [22]. In addition, the correction for medical school affiliation corrects not only for differences in patient severity but also for treatment by and supervision of doctors and nurses in training. The use of these adjustment factors has also been questioned [9]. Moreover, not controlling for confounding variables such as facility type and patient acuity in the data will tend to mask any observed correlation with the HCAHPS survey data and not augment it. Thus, to evaluate these confounding factors, we performed a linear regression analysis with the SIR as the outcome to determine whether the variables in the HCAHPS survey would maintain significance.

## Results

Our study revealed that there were a total of 10,866 observed CLABSI cases and 9,543,765 CLABSI central line days reported by 1987 analyzed facilities. Table 1 describes the independent and dependent variables in our study, and the questions that inform the composite measures for communication with nurses and responsiveness of hospital staff.

Table 2 displays descriptive statistics of the dependent and independent variables. Three independent variables were assessed in the model. Because ZIP regression modeling performs regression analysis on the certain zeros versus nonzeros (logistic) and on nonzero count data (Poisson), we present the dependent variable of observed CLABSI cases as including the zeros (mean = 5.47) and excluding the zeros (mean = 7.15). There were an average of 4803 CLABSI central line days across all 1987 hospitals. Among the independent variables, 12.13% of surveyed patients reported that they sometimes or never received help as soon as they needed, 10.25% reported that their room and bathroom were sometimes or never clean, and 5.7% reported that their nurses sometimes or never communicated well (Table 2).

Table 3 shows univariate associations between the continuous independent variables and a calculated CLABSI rate, a proxy for the dependent variable. All continuous independent variables were significantly (p<0.0001) associated with the proxy outcome and were further tested in the ZIP regression model, with responsiveness of hospital staff (r = 0.18135) and communication with nurses (r = 0.16314) having the greatest correlation with the proxy outcome. All three continuous independent variables were also highly intercorrelated, meaning that patients usually respond similarly to various questions; i.e., if a patient reports having poor hospital staff responsiveness, s/he is more likely to also report that the hospital environment was not clean.

**Table 3:** Pearson correlation coefficients between CLABSI rate and independent variables.

*n = 1987 hospitals*					
Independent Variables, correlation coefficient (*p-value*)	Percent of patients who reported that they “sometimes” or “never” received help as soon as they wanted	Percent of patients who reported that their room and bathroom were “sometimes” or “never” clean	Percent of patients who reported that their nurses “sometimes” or “never” communicated well	CLABSI Rate (1 infection/10,000 central line days)	CLABSI SIR
Percent of patients who reported that they “sometimes” or “never” received help as soon as they wanted	-	0.67630 (*p<0.0001*)	0.89089 *(p<0.0001)*	0.18135 *(p<0.0001)*	0.16070 *(p<0.0001)*
Percent of patients who reported that their room and bathroom were “sometimes” or “never” clean	-	-	0.67965 *(p<0.0001)*	0.11177 *(p<0.0001)*	0.08600 *(p<0.0001)*
Percent of patients who reported that their nurses “sometimes” or “never” communicated well	-	-	-	0.16314 *(p<0.0001)*	0.15246 *(p<0.0001)*

Further bivariate analysis with variables tested one at a time against the dependent in a ZIP regression model is in Table 4; all variables were independently significantly (p<0.0001) associated with the outcome CLABSI cases per hospital.

**Table 4:** Bivariate analysis between observed CLABSI cases and independent variables.

*n = 1987 hospitals*						
	Unadjusted Estimated Rate Ratio (95% CI)	Lower 95% Confidence Interval	Upper 95% Confidence Interval	Wald Chi-Square	p-value	AIC
*Independent variables*						
Percent of patients who reported that they “sometimes” or “never” received help as soon as they wanted (for every 5 unit increase)	1.16	1.14	1.19	212.35	<0.0001	10463
Percent of patients who reported that their room and bathroom were “sometimes” or “never” clean (for every 5 unit increase)	1.11	1.08	1.14	60.96	<0.0001	10601
Percent of patients who reported that their nurses “sometimes” or “never” communicated well (for every 5 unit increase)	1.25	1.21	1.30	165.06	<0.0001	10507

*Note:* Variables run one at a time independently against dependent with offset term being (ln)central line days, and the zeromodel only including the variable central line days.

Zero-inflated Poisson distribution used.

The final ZIP regression model in Table 5 shows that as the percent of patients who reported that they “sometimes” or “never” received help as soon as they wanted increases by 5 units (e.g., 5% to 10%), the rate of infections significantly increases by a factor of 1.16 (every 5 unit increase, estimated RR = 1.16, 95% CI 1.14–1.19). Neither of the variables communication with nurses nor cleanliness of hospital environment reached significance in the final model, and had no impact on the main effect size of staff responsiveness when added or removed, so were not included in the final model. The final model has an AIC of 10,463. Finally, CLABSI central line days was significantly associated with a decreased odds (every 1000 day increase, estimated OR = 0.71, 95% CI 0.65–0.77) of hospitals having zero CLABSI cases. That is, for every 1000 day increase in reported central line days, the odds that a hospital would report zero CLABSIs decreases by a factor of 0.71.

**Table 5:** Final zero-inflated Poisson regression model for US acute care hospitals for observed CLABSI cases by independent variables.

*n = 1987 hospitals*
*non-zero observations = 1522*
*zero observations = 465*
*Dependent variable = observed CLABSI cases by hospital, with zip distribution*
*offset = (ln)central line days*
*zero model = central line days, with logit link function*

*Non Zero Model*					
Independent variables	Adjusted Estimated Rate Ratio	Lower 95% Confidence Interval	Upper 95% Confidence Interval	Wald Chi-Square	p-value
Percent of patients who reported that they “sometimes” or “never” received help as soon as they wanted (for every 5 unit increase)	1.16	1.14	1.19	212.35	<0.0001

Model AIC 10463					
*Certain Zero Model (0 cases vs at least one case)*	Estimated Odds Ratio	Lower 95% Confidence Interval	Upper 95% Confidence Interval	Wald Chi-Square	p-value
CLABSI central line days (for every 1000 day increase)	0.71	0.65	0.77	61	<0.0001

The linear regression analysis using the SIR found a similar degree of correlation between the HCAHPS survey data and a facility's SIR for CLABSIs. The final model only included the staff responsiveness variable, as the addition of the other variables did not change the effect size of staff responsiveness. Thus, the final model revealed that staff responsiveness was significantly associated with the dependent variable, SIR (estimated *b*-coefficient = 0.01973, SE 0.00272, p<0.0001, CI 0.0144, 0.0251). That is, for every 5 unit increase in the percent of patients who reported that they “sometimes” or “never” received help as soon as they wanted, the SIR increased by 0.09865 units (0.01973*5). The R-Square value for the linear regression model was 0.0258.

The analysis of the SIRs for the upper and lower quartile of the 2010 aggregate state CDC CLABSI data found no significant difference observed between low-reporting states and high-reporting states. The SIR for states that had less than or equal to 20.7% of facilities reporting (N = 11) was 0.678, compared to an SIR of 0.7155 for states that had equal to or greater than 64.9% of facilities reporting (N = 12). This is evidence that state mandatory reporting initiatives was not a significant variable in the validity of the reported rate of CLABSIs.

## Discussion

The two variables which had the highest independent correlation with CLABSIs were communication between the nurse and the patient and hospital responsiveness to patients. Both of these variables would be expected to be highly dependent upon staffing levels and the quality of a facility's staff.

It has been observed that as staffing is cut in a facility the overall rate of healthcare acquired conditions including infections, increases [11], [31]. Prevention of CLABSIs has also been observed to be dependent upon the nursing staff; a low nurse-to-patient ratio is associated with an increase in CLABSIs [12]. In addition to nurse staffing, a recent study by Cimiotti et al (2012) found that nurse burnout was associated with an increase in urinary tract infections and surgical site infections even after controlling for nurse staffing, which became nonsignificant in their final model [13].

Although reductions in nurse staffing is associated with a higher level of adverse events, an Agency for Healthcare Research and Quality (AHRQ) study found that this is not always causal, but reflective of the institution's commitment to quality care and that deficiencies may exist in other areas [32]. This represents a problem with the facility's patient safety culture and may be indicative of a cost-driven rather than a patient-centered organization. Unfortunately in our healthcare system, cost-driven organizations appear to be becoming more common [33] and one of the main ways to increase net income or profit is to cut staffing [11].

Finally, though all the tested variables were independently significantly associated with CLABSIs, only one maintained significance in the final model: staff responsiveness. Cleanliness of the hospital environment and poor nurse communication with patients, as reported by patients, were not significant in the final model, mainly because of the high intercorrelation between the patient survey variables. Thus, patients who responded negatively in one dimension of the survey were likely to respond similarly across other dimensions.

This is telling; hospitals that have a poor patient safety culture may not be doing well across multiple dimensions and are more likely to be a poorer performing hospital in general. Another possibility is that patients who had one poor experience with staff responsiveness may be responding similarly across other dimensions.

Nevertheless, our study found that poor staff responsiveness was significantly associated with an increased risk in CLABSIs, and that this finding was robust and maintained a similar effect size even when all variables were included in the model and when an additional analysis was performed using the SIR as the dependent variable. The implications of this study are broad, and can help hospital decision makers evaluate the culture of safety at their facility. This study also supports the published literature that the working environment and appropriate staffing levels are important in fostering this culture [14], [15], [34].

### Strengths and limitations

This study is of limited scope as we were not able to account for all possible CLABSI risk factors such as bed size, teaching institution, or bed size because these were not publicly available, and we were unable to correlate these to all of the hundreds of metrics currently used to measure hospital quality. However, we found similar results when we repeated our analysis using the SIR as the outcome, which is adjusted for patient mix and facility characteristics.

Another limitation is that CLABSI reporting is required only for ICUs, while patients' survey responses were hospital wide. ICU-only patient responses may be different than other patient responses. However, this type of bias will tend to mask significance. Because significant correlation was observed between the survey questions and CLABSI rates, one of two conclusions may be drawn: That the correlation between the ICU surveys and CLABSIs was so strong as to override the effects of the influence of the other surveys; or more likely that the observed CLABSIs rates are related to the overall safety culture found at a facility.

Also, patient survey responses are susceptible to recall bias (e.g., not remembering the cleanliness of a room or bathroom) and selection or sampling bias (e.g., only patients that had very good or bad experiences responded). However, patient web-based reporting systems have been shown to be predictive of facility quality [35]. In addition, any bias introduced with recall bias should tend to mask not augment significance. Sampling bias where negative results are reported more frequently will not negate the validity of the differentiation between facilities with good and poor patient evaluations.

Conversely, the strength of our study is that it is consistent with and adds to the extensive knowledge base regarding the importance of a facility's culture of safety, both among its staff and administration.

## Conclusions

This study adds to the literature on the importance of a culture of safety in the prevention of healthcare associated infections. This study found overall hospital staff responsiveness, as assessed by recently discharged inpatients, was associated with an increased reported level of ICU CLABSIs. Poor staff responsiveness is a likely surrogate metric for staff working environments and workloads maintained by facilities. The association of poor staff responsiveness with an increased level of CLABSIs may not be a direct cause but a symptom of hospitals with a multitude of problems, including a poor culture of safety, a poor working environment, high staff work loads, and nurse burnout. Finally, when confronted with poor patient survey scores, hospital decision makers need to regard these as a possible symptom of safety problems at multiple levels in their delivery system and not just focus on improving isolated metrics.
